# 3-[(*E*)-2-Chloro-3,3,3-trifluoro­prop-1-en-1-yl]-*N*-(2-chloro­phen­yl)-2,2-dimethyl­cyclo­propane-1-carboxamide

**DOI:** 10.1107/S1600536810050634

**Published:** 2010-12-11

**Authors:** Dong-Qing Liu, Fan-Yong Yan, Yun-Ying Gao, Lei Guo, Zi Kong

**Affiliations:** a School of Materials Science and Engineering, Tianjin Polytechnic University, Tianjin 300160, People’s Republic of China; b School of Materials and Chemical Engineering, Tianjin Polytechnic University, Tianjin 300160, People’s Republic of China

## Abstract

In the title compound, C_15_H_14_Cl_2_F_3_NO, synthesized by the reaction of 3-[(*E*)-2-chloro-3,3,3-trifluoro­prop-1-en­yl]-2,2-dimethyl­cyclo­propane­carb­oxy­lic acid and 2-chloro­aniline, the aromatic ring makes a dihedral angle of 76.7 (3)° with the plane of the cyclo­propane ring. In the crystal, inter­molecular N—H⋯O hydrogen bonds link the mol­ecules into chains running along the *b* axis.

## Related literature

The title compound is an inter­mediate for tefluthrinn (2,3,5,6-tetrafluoro-4-methylbenzyl(1*RS*,3*RS*)-3-[(*Z*)-2-chloro-3,3,3-trifluoroprop-1-enyl]-2,2-dimethylcyclopropanecarboxylate), an insecticide controlling a wide range of soil insect pests, see: Punja (1981 ▶). For the preparation of the title compound, see Liu & Yan (2007 ▶). For a related structure, see: Yan *et al.* (2010 ▶).
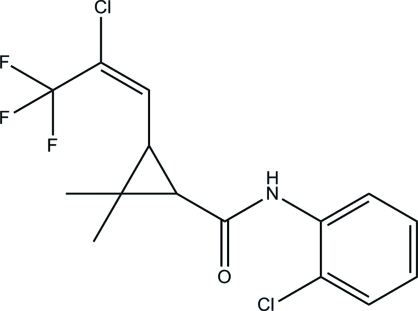


## Experimental

### 

#### Crystal data

C_15_H_14_Cl_2_F_3_NO
*M* *_r_* = 352.17
Orthorhombic, 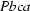

*a* = 18.454 (4) Å
*b* = 9.3350 (19) Å
*c* = 18.981 (4) Å
*V* = 3269.7 (11) Å^3^
*Z* = 8
Mo *K*α radiation
μ = 0.43 mm^−1^
*T* = 113 K
0.40 × 0.06 × 0.06 mm

#### Data collection

Rigaku Saturn CCD area-detector diffractometer
Absorption correction: multi-scan (*CrystalClear*; Rigaku/MSC, 2005 ▶) *T*_min_ = 0.848, *T*_max_ = 0.975
28276 measured reflections
3873 independent reflections
3325 reflections with *I* > 2σ(*I*)
*R*_int_ = 0.045

#### Refinement

*R*[*F* ^2^ > 2σ(*F* ^2^)] = 0.044
*wR*(*F* ^2^) = 0.115
*S* = 1.08
3873 reflections
206 parameters
H atoms treated by a mixture of independent and constrained refinement
Δρ_max_ = 0.25 e Å^−3^
Δρ_min_ = −0.37 e Å^−3^

### 

Data collection: *CrystalClear* (Rigaku/MSC, 2005 ▶); cell refinement: *CrystalClear*; data reduction: *CrystalClear*; program(s) used to solve structure: *SHELXS97* (Sheldrick, 2008 ▶); program(s) used to refine structure: *SHELXL97* (Sheldrick, 2008 ▶); molecular graphics: *SHELXTL* (Sheldrick, 2008 ▶); software used to prepare material for publication: *SHELXTL*.

## Supplementary Material

Crystal structure: contains datablocks I, global. DOI: 10.1107/S1600536810050634/bt5407sup1.cif

Structure factors: contains datablocks I. DOI: 10.1107/S1600536810050634/bt5407Isup2.hkl

Additional supplementary materials:  crystallographic information; 3D view; checkCIF report

## Figures and Tables

**Table 1:** Hydrogen-bond geometry (Å, °)

*D*—H⋯*A*	*D*—H	H⋯*A*	*D*⋯*A*	*D*—H⋯*A*
N1—H1⋯O1^i^	0.81 (2)	2.26 (2)	3.0415 (19)	162 (2)

Symmetry code: (i) 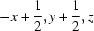.

**Fig. 1.**
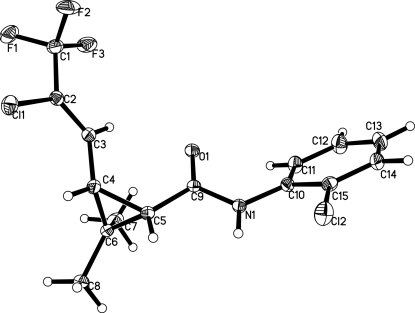
The molecular structure of the title compound, drawn with 30% probability ellipsoids. H atoms are drawn as spheres of arbitrary radius.

## supplementary crystallographic information

### Comment

3-((*E*)-2-chloro-3,3,3-trifluoroprop-1-enyl)-2,2-dimethyl cyclopropanecarboxylic acid is a very important intermediate for tefluthrin, an important insecticide controlling a wide range of soil insect pests in maize, sugar beet, and other crops (Punja, 1981).   The title compound may  show some insecticide activity. The present X-ray crystal structure analysis was undertaken in order to study the stereochemistry and crystal packing of the title compound. The dihedral angle between the aromatic ring and the cyclopropane groug is 76.7 (3)°. An N-H···O hydrogen bond links the molecules to chains running along the b-axis.

### Experimental

The title compound was prepared according to the method of Liu & Yan (2007). The product was recrystallized from methanol and ethyl acetate (5:1) over 5 d at ambient temperature, gave colourless single crystals of (*E*)-3-(2-chloro-3,3,3-trifluoroprop-1-en-1-yl)-*N*-(2-chlorophenyl)-2,2- dimethylcyclopropanecarboxamide, suitable for X-ray analysis.

### Refinement

H atoms were positioned geometrically with C—H = 0.93–0.98 Å and refined using riding model with *U*_iso_(H) = 1.2Ueq(carrier). The amiino H atom was located from a difference map and freely refined.

### Crystal data

**Table:**

C_15_H_14_Cl_2_F_3_NO	*D*_x_ = 1.431 Mg m^−^^3^
*M**_r_* = 352.17	Melting point: 124 K
Orthorhombic, *P**b**c**a*	Mo *K*α radiation, λ = 0.71073 Å
*a* = 18.454 (4) Å	Cell parameters from 8396 reflections
*b* = 9.3350 (19) Å	θ = 2.2–27.2°
*c* = 18.981 (4) Å	µ = 0.43 mm^−^^1^
*V* = 3269.7 (11) Å^3^	*T* = 113 K
*Z* = 8	Block, colorless
*F*(000) = 1440	0.40 × 0.06 × 0.06 mm

### Data collection

**Table:**

Rigaku Saturn CCD area-detector diffractometer	3873 independent reflections
Radiation source: rotating anode	3325 reflections with *I* > 2σ(*I*)
confocal	*R*_int_ = 0.045
Detector resolution: 7.31 pixels mm^-1^	θ_max_ = 27.9°, θ_min_ = 2.2°
ω and φ scans	*h* = −22→24
Absorption correction: multi-scan (*CrystalClear*; Rigaku/MSC, 2005)	*k* = −10→12
*T*_min_ = 0.848, *T*_max_ = 0.975	*l* = −24→24
28276 measured reflections	

### Refinement

**Table:**

Refinement on *F*^2^	Secondary atom site location: difference Fourier map
Least-squares matrix: full	Hydrogen site location: inferred from neighbouring sites
*R*[*F*^2^ > 2σ(*F*^2^)] = 0.044	H atoms treated by a mixture of independent and constrained refinement
*wR*(*F*^2^) = 0.115	*w* = 1/[σ^2^(*F*_o_^2^) + (0.0648*P*)^2^ + 0.6506*P*] where *P* = (*F*_o_^2^ + 2*F*_c_^2^)/3
*S* = 1.08	(Δ/σ)_max_ = 0.001
3873 reflections	Δρ_max_ = 0.25 e Å^−^^3^
206 parameters	Δρ_min_ = −0.37 e Å^−^^3^
0 restraints	Extinction correction: *SHELXL97* (Sheldrick, 2008), Fc^*^=kFc[1+0.001xFc^2^λ^3^/sin(2θ)]^-1/4^
Primary atom site location: structure-invariant direct methods	Extinction coefficient: 0.0143 (13)

### Special details

**Table:**

Geometry. All e.s.d.'s (except the e.s.d. in the dihedral angle between two l.s. planes) are estimated using the full covariance matrix. The cell e.s.d.'s are taken into account individually in the estimation of e.s.d.'s in distances, angles and torsion angles; correlations between e.s.d.'s in cell parameters are only used when they are defined by crystal symmetry. An approximate (isotropic) treatment of cell e.s.d.'s is used for estimating e.s.d.'s involving l.s. planes.
Refinement. Refinement of *F*^2^ against ALL reflections. The weighted *R*-factor *wR* and goodness of fit *S* are based on *F*^2^, conventional *R*-factors *R* are based on *F*, with *F* set to zero for negative *F*^2^. The threshold expression of *F*^2^ > σ(*F*^2^) is used only for calculating *R*-factors(gt) *etc*. and is not relevant to the choice of reflections for refinement. *R*-factors based on *F*^2^ are statistically about twice as large as those based on *F*, and *R*- factors based on ALL data will be even larger.

### Fractional atomic coordinates and isotropic or equivalent isotropic displacement parameters (Å^2^)

**Table:**

	*x*	*y*	*z*	*U*_iso_*/*U*_eq_	
Cl1	0.15849 (2)	0.37167 (5)	0.05290 (2)	0.03411 (15)	
Cl2	0.44326 (2)	0.57979 (5)	0.35812 (2)	0.03963 (16)	
F1	0.08112 (7)	0.08899 (12)	0.07614 (7)	0.0458 (3)	
F2	0.19548 (7)	0.06413 (12)	0.09253 (7)	0.0490 (3)	
F3	0.12256 (7)	0.05370 (11)	0.18026 (6)	0.0440 (3)	
O1	0.20892 (6)	0.32062 (12)	0.32957 (6)	0.0293 (3)	
N1	0.28640 (7)	0.50060 (15)	0.35929 (7)	0.0237 (3)	
C1	0.13526 (10)	0.12238 (19)	0.12027 (10)	0.0320 (4)	
C2	0.14360 (8)	0.27922 (17)	0.13093 (8)	0.0251 (3)	
C3	0.14182 (9)	0.34166 (18)	0.19383 (9)	0.0275 (3)	
H3	0.1325	0.2838	0.2327	0.033*	
C4	0.15340 (10)	0.49437 (18)	0.20662 (9)	0.0313 (4)	
H4	0.1598	0.5517	0.1638	0.038*	
C5	0.19808 (9)	0.54696 (17)	0.27041 (9)	0.0281 (4)	
H5	0.2278	0.6318	0.2611	0.034*	
C6	0.11674 (10)	0.57294 (18)	0.26648 (10)	0.0346 (4)	
C7	0.06647 (10)	0.4966 (2)	0.31580 (12)	0.0456 (5)	
H7A	0.0198	0.4862	0.2940	0.068*	
H7B	0.0616	0.5510	0.3585	0.068*	
H7C	0.0858	0.4037	0.3266	0.068*	
C8	0.09538 (13)	0.7269 (2)	0.24999 (14)	0.0590 (7)	
H8A	0.0491	0.7279	0.2269	0.089*	
H8B	0.1311	0.7692	0.2197	0.089*	
H8C	0.0925	0.7807	0.2930	0.089*	
C9	0.23019 (8)	0.44437 (16)	0.32159 (8)	0.0232 (3)	
C10	0.33010 (8)	0.42237 (17)	0.40673 (8)	0.0236 (3)	
C11	0.30110 (10)	0.31756 (19)	0.45095 (9)	0.0316 (4)	
H11	0.2515	0.3001	0.4505	0.038*	
C12	0.34543 (11)	0.2394 (2)	0.49541 (11)	0.0416 (5)	
H12	0.3253	0.1699	0.5246	0.050*	
C13	0.41935 (11)	0.2637 (2)	0.49691 (11)	0.0418 (5)	
H13	0.4489	0.2092	0.5262	0.050*	
C14	0.44927 (10)	0.3692 (2)	0.45467 (10)	0.0342 (4)	
H14	0.4988	0.3872	0.4560	0.041*	
C15	0.40456 (9)	0.44771 (17)	0.41045 (8)	0.0269 (3)	
H1	0.2974 (11)	0.583 (2)	0.3510 (11)	0.032 (5)*	

### Atomic displacement parameters (Å^2^)

**Table:**

	*U*^11^	*U*^22^	*U*^33^	*U*^12^	*U*^13^	*U*^23^
Cl1	0.0350 (2)	0.0409 (3)	0.0265 (2)	−0.00101 (17)	0.00151 (16)	0.00143 (16)
Cl2	0.0316 (2)	0.0479 (3)	0.0394 (3)	−0.01434 (18)	0.00113 (18)	0.0092 (2)
F1	0.0431 (7)	0.0421 (6)	0.0521 (7)	−0.0071 (5)	−0.0118 (5)	−0.0169 (5)
F2	0.0400 (6)	0.0392 (7)	0.0680 (8)	0.0103 (5)	0.0079 (6)	−0.0207 (6)
F3	0.0638 (8)	0.0226 (5)	0.0454 (6)	−0.0048 (5)	0.0009 (6)	−0.0010 (5)
O1	0.0333 (6)	0.0187 (6)	0.0361 (6)	−0.0026 (4)	−0.0067 (5)	0.0029 (5)
N1	0.0257 (7)	0.0187 (7)	0.0266 (7)	−0.0020 (5)	−0.0024 (5)	0.0029 (5)
C1	0.0296 (9)	0.0295 (9)	0.0369 (9)	0.0017 (7)	−0.0015 (7)	−0.0092 (7)
C2	0.0225 (7)	0.0246 (8)	0.0281 (7)	0.0002 (6)	−0.0006 (6)	0.0002 (6)
C3	0.0323 (8)	0.0223 (8)	0.0279 (8)	0.0016 (6)	−0.0043 (6)	0.0002 (6)
C4	0.0443 (10)	0.0204 (8)	0.0292 (8)	−0.0010 (7)	−0.0113 (7)	−0.0002 (6)
C5	0.0339 (9)	0.0186 (7)	0.0318 (8)	−0.0012 (6)	−0.0089 (7)	0.0004 (6)
C6	0.0380 (10)	0.0258 (9)	0.0401 (10)	0.0094 (7)	−0.0186 (8)	−0.0117 (7)
C7	0.0307 (9)	0.0539 (13)	0.0521 (12)	0.0106 (9)	−0.0046 (8)	−0.0234 (10)
C8	0.0721 (15)	0.0283 (10)	0.0766 (16)	0.0212 (10)	−0.0477 (13)	−0.0202 (10)
C9	0.0243 (7)	0.0195 (7)	0.0257 (7)	0.0034 (6)	−0.0004 (6)	−0.0026 (6)
C10	0.0232 (8)	0.0250 (8)	0.0225 (7)	0.0012 (6)	−0.0010 (6)	−0.0002 (6)
C11	0.0300 (8)	0.0332 (9)	0.0316 (8)	−0.0042 (7)	−0.0022 (7)	0.0078 (7)
C12	0.0437 (11)	0.0382 (11)	0.0429 (10)	−0.0058 (8)	−0.0092 (8)	0.0168 (8)
C13	0.0429 (10)	0.0370 (10)	0.0454 (10)	0.0061 (8)	−0.0147 (9)	0.0081 (8)
C14	0.0251 (8)	0.0380 (10)	0.0394 (9)	0.0038 (7)	−0.0053 (7)	−0.0040 (8)
C15	0.0273 (8)	0.0263 (8)	0.0270 (8)	−0.0014 (6)	0.0014 (6)	−0.0015 (6)

### Geometric parameters (Å, °)

**Table:**

Cl1—C2	1.7360 (17)	C6—C7	1.498 (3)
Cl2—C15	1.7368 (17)	C6—C8	1.523 (3)
F1—C1	1.340 (2)	C7—H7A	0.9600
F2—C1	1.345 (2)	C7—H7B	0.9600
F3—C1	1.328 (2)	C7—H7C	0.9600
O1—C9	1.2294 (19)	C8—H8A	0.9600
N1—C9	1.365 (2)	C8—H8B	0.9600
N1—C10	1.412 (2)	C8—H8C	0.9600
N1—H1	0.81 (2)	C10—C11	1.396 (2)
C1—C2	1.486 (2)	C10—C15	1.396 (2)
C2—C3	1.329 (2)	C11—C12	1.383 (3)
C3—C4	1.462 (2)	C11—H11	0.9300
C3—H3	0.9300	C12—C13	1.383 (3)
C4—C6	1.512 (3)	C12—H12	0.9300
C4—C5	1.545 (2)	C13—C14	1.385 (3)
C4—H4	0.9800	C13—H13	0.9300
C5—C9	1.487 (2)	C14—C15	1.387 (2)
C5—C6	1.522 (2)	C14—H14	0.9300
C5—H5	0.9800		
			
C9—N1—C10	124.70 (14)	C6—C7—H7A	109.5
C9—N1—H1	117.0 (15)	C6—C7—H7B	109.5
C10—N1—H1	118.1 (15)	H7A—C7—H7B	109.5
F3—C1—F1	106.98 (15)	C6—C7—H7C	109.5
F3—C1—F2	106.64 (15)	H7A—C7—H7C	109.5
F1—C1—F2	106.10 (14)	H7B—C7—H7C	109.5
F3—C1—C2	112.16 (14)	C6—C8—H8A	109.5
F1—C1—C2	113.04 (15)	C6—C8—H8B	109.5
F2—C1—C2	111.48 (15)	H8A—C8—H8B	109.5
C3—C2—C1	123.50 (16)	C6—C8—H8C	109.5
C3—C2—Cl1	123.52 (13)	H8A—C8—H8C	109.5
C1—C2—Cl1	112.95 (12)	H8B—C8—H8C	109.5
C2—C3—C4	124.99 (16)	O1—C9—N1	122.64 (14)
C2—C3—H3	117.5	O1—C9—C5	123.94 (14)
C4—C3—H3	117.5	N1—C9—C5	113.42 (13)
C3—C4—C6	122.17 (16)	C11—C10—C15	117.76 (15)
C3—C4—C5	121.21 (14)	C11—C10—N1	121.81 (15)
C6—C4—C5	59.72 (11)	C15—C10—N1	120.43 (14)
C3—C4—H4	114.3	C12—C11—C10	120.65 (16)
C6—C4—H4	114.3	C12—C11—H11	119.7
C5—C4—H4	114.3	C10—C11—H11	119.7
C9—C5—C6	121.83 (15)	C13—C12—C11	120.64 (18)
C9—C5—C4	121.32 (14)	C13—C12—H12	119.7
C6—C5—C4	59.07 (11)	C11—C12—H12	119.7
C9—C5—H5	114.5	C12—C13—C14	119.84 (17)
C6—C5—H5	114.5	C12—C13—H13	120.1
C4—C5—H5	114.5	C14—C13—H13	120.1
C7—C6—C4	121.05 (15)	C13—C14—C15	119.29 (17)
C7—C6—C5	120.27 (15)	C13—C14—H14	120.4
C4—C6—C5	61.21 (11)	C15—C14—H14	120.4
C7—C6—C8	114.65 (18)	C14—C15—C10	121.78 (16)
C4—C6—C8	114.78 (18)	C14—C15—Cl2	118.48 (13)
C5—C6—C8	114.56 (17)	C10—C15—Cl2	119.74 (13)
			
F3—C1—C2—C3	2.9 (2)	C9—C5—C6—C8	144.09 (17)
F1—C1—C2—C3	123.98 (18)	C4—C5—C6—C8	−105.95 (19)
F2—C1—C2—C3	−116.59 (19)	C10—N1—C9—O1	5.5 (2)
F3—C1—C2—Cl1	−178.82 (11)	C10—N1—C9—C5	−174.29 (14)
F1—C1—C2—Cl1	−57.75 (18)	C6—C5—C9—O1	50.0 (2)
F2—C1—C2—Cl1	61.67 (17)	C4—C5—C9—O1	−20.8 (2)
C1—C2—C3—C4	176.79 (16)	C6—C5—C9—N1	−130.28 (15)
Cl1—C2—C3—C4	−1.3 (2)	C4—C5—C9—N1	159.01 (15)
C2—C3—C4—C6	148.84 (17)	C9—N1—C10—C11	−38.7 (2)
C2—C3—C4—C5	−139.55 (18)	C9—N1—C10—C15	141.04 (16)
C3—C4—C5—C9	−0.7 (3)	C15—C10—C11—C12	−1.7 (3)
C6—C4—C5—C9	110.81 (18)	N1—C10—C11—C12	178.02 (17)
C3—C4—C5—C6	−111.54 (19)	C10—C11—C12—C13	0.0 (3)
C3—C4—C6—C7	0.1 (2)	C11—C12—C13—C14	1.4 (3)
C5—C4—C6—C7	−109.91 (18)	C12—C13—C14—C15	−1.1 (3)
C3—C4—C6—C5	109.97 (17)	C13—C14—C15—C10	−0.7 (3)
C3—C4—C6—C8	−144.45 (17)	C13—C14—C15—Cl2	179.85 (14)
C5—C4—C6—C8	105.58 (17)	C11—C10—C15—C14	2.0 (2)
C9—C5—C6—C7	1.2 (2)	N1—C10—C15—C14	−177.69 (15)
C4—C5—C6—C7	111.14 (18)	C11—C10—C15—Cl2	−178.50 (13)
C9—C5—C6—C4	−109.96 (17)	N1—C10—C15—Cl2	1.8 (2)

### Hydrogen-bond geometry (Å, °)

**Table:**

*D*—H···*A*	*D*—H	H···*A*	*D*···*A*	*D*—H···*A*
N1—H1···O1^i^	0.81 (2)	2.26 (2)	3.0415 (19)	162 (2)

Symmetry codes: (i) −*x*+1/2, *y*+1/2, *z*.

## References

[bb1] Liu, D.-Q. & Yan, F.-Y. (2007). *Acta Cryst.* E**63**, o4202.

[bb2] Punja, N. (1981). Eur. Patent EP 0031199.

[bb3] Rigaku/MSC (2005). *CrystalClear* Rigaku/MSC, The Woodlands, Texas, USA.

[bb4] Sheldrick, G. M. (2008). *Acta Cryst.* A**64**, 112–122. 10.1107/S0108767307043930 18156677

[bb5] Yan, F.-Y., Liu, D.-Q., Wen, J.-Y., Gao, Y.-Y. & Li, A.-M. (2011). *Acta Cryst.* E**67**, o60. 10.1107/S1600536810050464 PMC3050188 21522771

